# Pulmonary ultrasound provides a more accurate assessment of pulmonary congestion than wet/dry lung weight in rats

**DOI:** 10.14814/phy2.70701

**Published:** 2025-12-10

**Authors:** André Timóteo Sapalo

**Affiliations:** ^1^ Division of Emergency Medicine, Department of Internal Medicine, Vascular Biology Laboratory, School of Medicine São Paulo University Ribeirão Preto SP Brazil; ^2^ HCRP‐USP Echocardiography Laboratory, Department of Clinical Medicine, FMRP‐USP, Ribeirão Preto Medical School University of São Paulo Ribeirão Preto SP Brazil

**Keywords:** Evans blue dye, pulmonary B‐lines echocardiography, pulmonary congestion, pulmonary ultrasound

## Abstract

Pulmonary congestion is a common complication in critically ill patients, but its quantification remains challenging. The wet‐to‐dry (W/D) lung weight ratio, widely used in experimental models, is a terminal and semi‐quantitative method. Lung ultrasonography (LU) may offer a non‐invasive and reproducible alternative for assessing pulmonary edema. To evaluate the accuracy and reproducibility of LU compared with the W/D ratio in a rat model of oleic acid–induced lung injury. Thirty Wistar‐Kyoto rats were randomized into injury (oleic acid, *n* = 15) and control (saline, *n* = 15) groups. Echocardiography and LU were performed at baseline and 1 h after infusion. B‐line scoring was independently analyzed by two echocardiographers, with reproducibility assessed by Bland–Altman plots. Evans blue dye evaluated vascular permeability. Oleic acid–treated rats showed significantly higher LU scores, B‐line counts, W/D ratios, and Evans blue levels than controls. LU demonstrated good diagnostic performance in detecting lung edema in this experimental model, with excellent inter‐ and intraobserver agreement confirming strong reproducibility. LU proved accurate, reproducible, and non‐terminal for detecting pulmonary congestion in rats, showing good agreement with traditional gravimetric measures and supporting its use for longitudinal assessment of pulmonary edema in experimental research.

## Graphical abstract

Graphical abstract illustrating the experimental rat model of oleic acid‐induced lung injury and the comparative assessment of pulmonary congestion using lung ultrasound (B‐lines), wet/dry lung weight ratio, and Evans Blue Dye quantification. Lung ultrasound demonstrated increased diagnostic accuracy and enables longitudinal monitoring of pulmonary congestion.

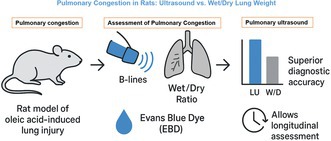

## 1 INTRODUCTION

Pulmonary co‐congestion is a clinical condition frequently observed in critically ill patients and is commonly associated with renal failure, acute respiratory distress syndrome (Wiedemann, 2008), acute heart failure, and septic shock (Santos et al., 2013). Consequently, the evaluation of extravascular lung water (EVLW) remains one of the major challenges in clinical practice. Accurate quantification of EVLW can offer critical information for optimizing treatment strategies, particularly in elderly patients (Ashton‐Cleary, 2013).

Several methods have been developed to assess EVLW, yet they are often limited by low specificity, invasiveness (Corradi et al., 2014), and other methodological constraints (Kuz'kov et al., 2007). Experimental models of pulmonary congestion have facilitated the development of new assessment techniques. The most widely used method in animal studies involves calculating the wet‐to‐dry lung weight ratio, typically performed post‐mortem. However, this method provides only a single time‐point measurement and may be subject to variability due to its semi‐quantitative nature.

Transthoracic lung ultrasound, in contrast, enables the real‐time detection of EVLW by visualizing multiple, diffuse B‐lines. While lung ultrasonography (LUS) is routinely applied in human and large‐animal medicine, its use for EVLW assessment in small animal models, such as rats, remains underexplored. Given that rats are commonly employed in experimental models of pulmonary pathology, including septic lung injury and acute lung injury (ALI), the ability to monitor EVLW non‐invasively and longitudinally would offer significant advantages. In particular, non‐terminal methods such as LUS could facilitate serial measurements and permit survival‐based studies, similar to the use of echocardiography in small animal research.

Based on this rationale, we hypothesized that LUS could reliably quantify pulmonary congestion in rats, providing comparable or superior accuracy to the conventional wet‐to‐dry lung weight ratio. This study was therefore designed to evaluate the level of agreement between LUS findings and standard gravimetric measurements in a rat model of oleic acid‐induced pulmonary congestion.

## 2 METHODS

### 2.1 Experimental animals

Thirty 10‐week‐old Wistar‐Kyoto (WKY) rats, of both sexes, were obtained from the Institute of Biomedical Sciences (ICB), University of São Paulo. The animals were housed in individually ventilated and temperature‐controlled cages under a 12‐h light/dark cycle, with ad libitum access to food and water. All rats received a commercially balanced rodent diet, provided in block/granule form (Nuvilab CR‐1, Nuvital Nutrientes, Brazil), offered ad libitum throughout the study. They remained under these controlled conditions until reaching 32 weeks of age. All experimental procedures were approved by the Institutional Animal Care and Use Committee of the Ribeirão Preto Medical School, University of São Paulo (Protocol No. 1042/2022), and were conducted in accordance with the ARRIVE 2.0 guidelines.

### 2.2 Study design

Animals were randomly assigned to one of two groups: control (*n* = 15) and lung injury (*n* = 15). Lung injury was induced via intravenous administration of oleic acid (OA), acquired from Sigma‐Aldrich® (Sigma‐Aldrich, Cat. No. O1008), and prepared as a 1:1 mixture with absolute ethanol, following the protocol described by Ma et al. (2015). Rats were positioned supine and infused via the caudal vein with OA at a dose of 9 μL/100 g over 5 min. Control animals received an equal volume of 0.9% normal saline (NS). Anesthesia depth was maintained with repeated intramuscular administration of ketamine and xylazine (0.10 U/L every 45 min), guided by cardiorespiratory and reflex monitoring.

### 2.3 Ultrasound

Cardiac function and pulmonary congestion parameters were assessed using transthoracic echocardiography (ECHO) and lung ultrasound (LUS) both at baseline and 1 hour after OA/NS injection. Imaging was performed using a Vevo® 2100 high‐resolution ultrasound system (VisualSonics Inc., Toronto, Canada) with a 30 MHz linear transducer. Animals were anesthetized with intramuscular ketamine (100 mg/kg) and xylazine (10 mg/kg), and the anterior and posterior thoracic regions were shaved.

For ECHO, rats were placed in the supine position; for LUS, they were positioned prone on a tilting platform. Body temperature and electrocardiogram (ECG) were continuously monitored under ambient air ventilation.

Echocardiographic measurements included: Left ventricular ejection fraction (LVEF), assessed via M‐mode and 2D B‐mode imaging; Fractional shortening (FS), calculated from M‐mode tracings; Diastolic function, evaluated using pulsed‐wave Doppler and tissue Doppler (TD) to measure early (E) and late (A) mitral inflow velocities and septal/lateral mitral annular velocities (e'); Left atrial (LA) size, measured using linear and volumetric methods (Rishniw method); Pulmonary artery pressure and resistance; Right ventricular (RV) function, assessed using tricuspid annular plane systolic excursion (TAPSE) and RV linear diameter.

### 2.4 Lung congestion assessment by lung ultrasound

**FIGURE 1:**
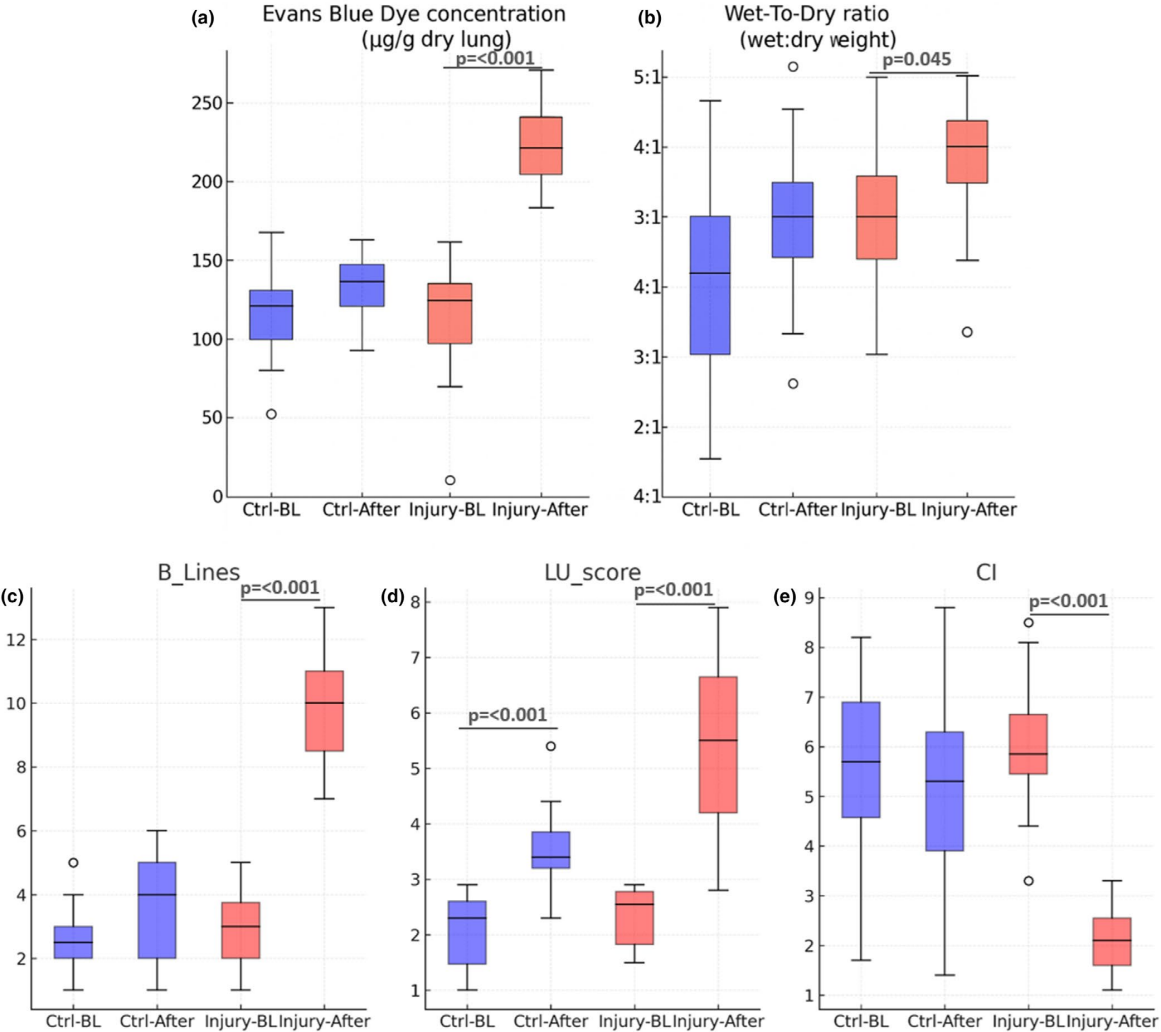
Boxplots of hemodynamic, echocardiographic, and pulmonary ultrasound parameters at baseline and after infusion in control (saline) and lung injury (oleic acid) groups. In all box‐and‐whisker plots, the central line represents the median, the box corresponds to the interquartile range (IQR), and the whiskers represent the minimum and maximum values within 1.5 × IQR. Outliers, when present, are displayed as individual points. Numerical *p*‐values are shown for all group comparisons. (a) concentrations increased significantly after oleic acid infusion but remained unchanged in controls. (b) W/D ratios increased in the lung injury group, with no change in controls. (c) The number of B‐lines rose significantly after oleic acid infusion, while controls were stable. (d) LU scores increased in the lung injury group but were unchanged in controls. (e) Cardiac index decreased significantly in oleic acid–treated animals, with no change in controls.

For LUS, bilateral scanning of the dorsal and lateral thoracic regions was performed. Each hemithorax was divided into two zones: (1) from the posterior axillary line to the scapular line, and (2) from the scapular to the paravertebral line. A total of four scans were obtained per rat in the vertical plane, each encompassing five to six intercostal spaces. The probe was moved in a vertical “up‐and‐down” fashion to visualize the full lung field, capturing approximately seven intercostal spaces per scan. Scan locations were marked to ensure consistent positioning (Figure 1).

### 2.5 B‐line scoring

At both baseline and 1 h post‐injection, A‐ and B‐lines were recorded. A‐lines appeared as horizontal reverberation artifacts, indicating normal lung aeration and received a score of 0. B‐lines were defined as vertical, hyperechoic, laser‐like artifacts originating from the pleural line, extending to the bottom of the screen without fading, and moving synchronously with lung sliding. In each intercostal space, B‐lines were counted (0–10), and their proportional occupation of the costal space was estimated and divided by 10 to calculate the B‐line score. Two experienced echocardiographers independently scored the images; discrepancies were resolved by consensus.

### 2.6 Assessment of pulmonary vascular leak

Pulmonary vascular permeability was assessed using Evans blue dye (EBD) extravasation, a well‐established marker of endothelial leak in experimental models. Elevated dye levels indicate increased permeability of the alveolocapillary barrier, which may precede or accompany pulmonary edema, but do not directly quantify total lung water accumulation. Evans blue dye was obtained from Sigma‐Aldrich® (Sigma‐Aldrich, Cat. No. E2129). One hour after OA/NS administration, eight rats from each group were injected intravenously with Evans blue (20 mg/kg) via the caudal vein. The dye circulated for 30 min to bind plasma albumin and distribute throughout the vasculature.

Subsequently, animals were euthanized under deep sedation induced by an intramuscular combination of ketamine and xylazine (0.1 mL per 100 g of body weight), and the pulmonary vasculature was flushed via the right ventricle with phosphate‐buffered saline (PBS) until the lungs were visibly blood‐free. Lungs were excised, weighed (wet weight), and processed for dye extraction. Lung tissues were homogenized in formamide and incubated at 60°C for 24 h. Supernatants were collected after centrifugation and analyzed spectrophotometrically at 620 nm. Evans blue concentrations were calculated against a standard curve and expressed as μg dye/g lung tissue. Elevated dye levels indicated increased vascular permeability and pulmonary congestion.

### 2.7 Measurement of wet‐to‐dry lung weight ratio

The lung wet‐to‐dry weight (W/D) ratio was used as an index of pulmonary edema. Seven animals per group were euthanized, and lungs were excised with the surrounding connective tissue removed. After blotting to remove surface moisture, the wet weight was recorded immediately. Lungs were then dried in an oven at 60°C for 48 h until a constant mass was achieved, after which the dry weight was recorded. The W/D ratio was calculated by dividing the wet weight by the dry weight. An increased ratio was indicative of higher water content and pulmonary congestion.

### 2.8 Reproducibility studies

Inter‐ and intraobserver reproducibility of the LU score was evaluated in the entire cohort. Both examiners were echocardiography technicians; one had formal training in lung ultrasonography, while the second received a 2‐h hands‐on training session from the first. The reliability of the B‐line score over time was assessed in 15 rats following saline infusion (control group) or oleic acid infusion (lung injury group).

### 2.9 Statistical analysis

Data normality was assessed using the Shapiro–Wilk test. Normally distributed continuous variables were presented as mean ± standard deviation (SD) and compared using independent *t*‐tests. Non‐normally distributed variables were reported as median (interquartile range, IQR) and analyzed using the Mann–Whitney *U* test. Categorical variables were expressed as counts (percentages) and compared using Fisher's exact test.

To assess the diagnostic performance of LUS and W/D ratio in detecting pulmonary congestion, EBD extravasation served as the reference standard. A threshold value of Evans blue concentration was used to classify lungs as congested or non‐congested. Receiver operating characteristic (ROC) curves were generated for both methods, and the area under the curve (AUC) was calculated. AUCs were compared using DeLong's test. Sensitivity, specificity, and predictive values were reported at optimal cutoff points. A two‐tailed *p*‐value <0.05 was considered statistically significant.

All statistical analyses were performed using GraphPad Prism (v9.1.2, GraphPad Software, San Diego, CA, USA), and ROC comparisons were conducted using MedCalc (MedCalc Software, Ostend, Belgium).

## 3 RESULTS

Several hemodynamic, echocardiographic, and lung ultrasound parameters differed significantly between groups. Animals in the lung‐injured group exhibited lower W/D ratios, increased RVD, elevated PASP and PVR, and a trend toward greater LVW. Diastolic function was impaired, as evidenced by lower E‐wave velocities, a trend toward a reduced E/A ratio, a higher tricuspid E/e' ratio, and significantly lower e' velocities. Lung ultrasound demonstrated a greater number of B‐lines and higher LU scores in the lung‐injured group, confirming increased extravascular lung water. In addition, both the inferior vena cava collapsibility index and TAPSE were reduced, indicating impaired ventricular systolic function (Table 1).

**TABLE 1:** Comparison of hemodynamic, echocardiographic, and pulmonary ultrasound parameters.

Variable	Control group (*n* = 15) (median [IQR])	Group lung injury (*n* = 15) (median [IQR])	Mann–Whitney *U*	*p*‐value
W/D ratio	3.4:1 [3.2–3.6]	4.0:1 [3.7–4.1]	64	0.045
FE_B	62.46 [58.03–63.96]	73.31 [70.33–76.21]	19	0.001
HR	216.00 [199.00–236.50]	231.00 [226.50–257.00]	68	0.067
SV	397.66 [334.75–450.60]	412.38 [364.67–450.29]	102	0.678
IVS	1.68 [1.35–1.88]	3.34 [2.96–3.60]	0	0.326
LVID	8.05 [7.80–8.61]	6.96 [6.67–7.21]	210	0.036
LVW	1.57 [1.34–1.90]	3.21 [3.06–3.45]	0	0.053
DRV	7.90 [6.55–8.70]	11.80 [10.85–12.80]	5,5	0.004
FS	43.48 [37.63–45.75]	42.55 [34.45–49.14]	115	0.933
Wave E	10.21 [8.76–10.92]	7.12 [6.76–8.39]	199.5	0.003
Wave A	3.40 [3.09–421.82]	3.82 [3.41–4.64]	8.5	0.237
E/A	2.23 [2.04–2.32]	1.97 [1.60–2.24]	1.4	0.140
e'	−34.55 [−35.63 to −31.85]	−30.23 [−34.01 to −25.91]	59	0.027
a'	‐25.91 [−26.45 to −23.21]	−24.830 [−25.910 to −23.210]	103	0.706
Lung ultrasound				
B‐Lines	4.00 [2.00–5.00]	10.000 [8.500–11.000]	0	0.032
LU score	3.40 [3.20–3.85]	5.50 [4.20–6.65]	27	0.001
Intravascular volume				
CI	5.30 [3.90–6.30]	2.10 [1.60–2.55]	202	0.002
TAPSE	9.00 [8.00–10.00]	4.00 [2.50–4.50]	224	0.039
Tricuspid E/e' ratio	2.10 [1.55–2.30]	5.50 [5.10–6.45]	0	0.004
PASP	7.00 [6.00–8.00]	11.00 [10.00–12.50]	4	0.016
PVR	2.10 [1.85–2.55]	4.60 [3.75–5.80]	0	0.047
EBD (μg/mL/dry lung wt)	167.45 [50.93–178.27]	223.15 [50.93–178.27]	29	<0.0001

Abbreviations: CI, collapsibility index; CI, distensibility index; DLA, left atrial end‐systolic transverse diameter; DLV, left ventricular end‐diastolic anteroposterior diameter; DRA, right atrial end‐systolic transverse diameter; EBD, Evans Blue dye extravasation; LU, lung ultrasound score; LVEF, left ventricular ejection fraction; LVO, left ventricular output; Mitral E/e' ratio, ratio between the peak early diastolic transmitral filling velocity and the peak early diastolic velocity of the lateral mitral annulus tissue; PASP, pulmonary artery systolic pressure; PVR, pulmonary vascular resistance; R‐time, reaction time; RVD, right ventricular end‐diastolic anteroposterior diameter; RVO, right ventricular output; RV‐PA coupling, right ventricular–pulmonary arterial coupling; TAPSE, tricuspid annular plane systolic excursion; Tricuspid E/e' ratio, ratio between the peak early diastolic transtricuspid filling velocity and the peak early diastolic velocity of the lateral tricuspid annulus tissue; W/D, lung wet‐to‐dry weight ratio.

In the control group, which received saline alone, the values of EBD, W/D ratio, B‐lines, LU score, and IVC collapsibility index showed only minor changes from baseline to post‐infusion, with no consistent evidence of pulmonary congestion. In contrast, animals in the lung injury group infused with oleic acid exhibited marked increases in EBD, W/D ratio, B‐lines, and LU score after infusion, indicating significant pulmonary fluid accumulation. These animals also demonstrated a pronounced reduction in the IVC collapsibility index compared with baseline (Figure 1).

**FIGURE 2:**
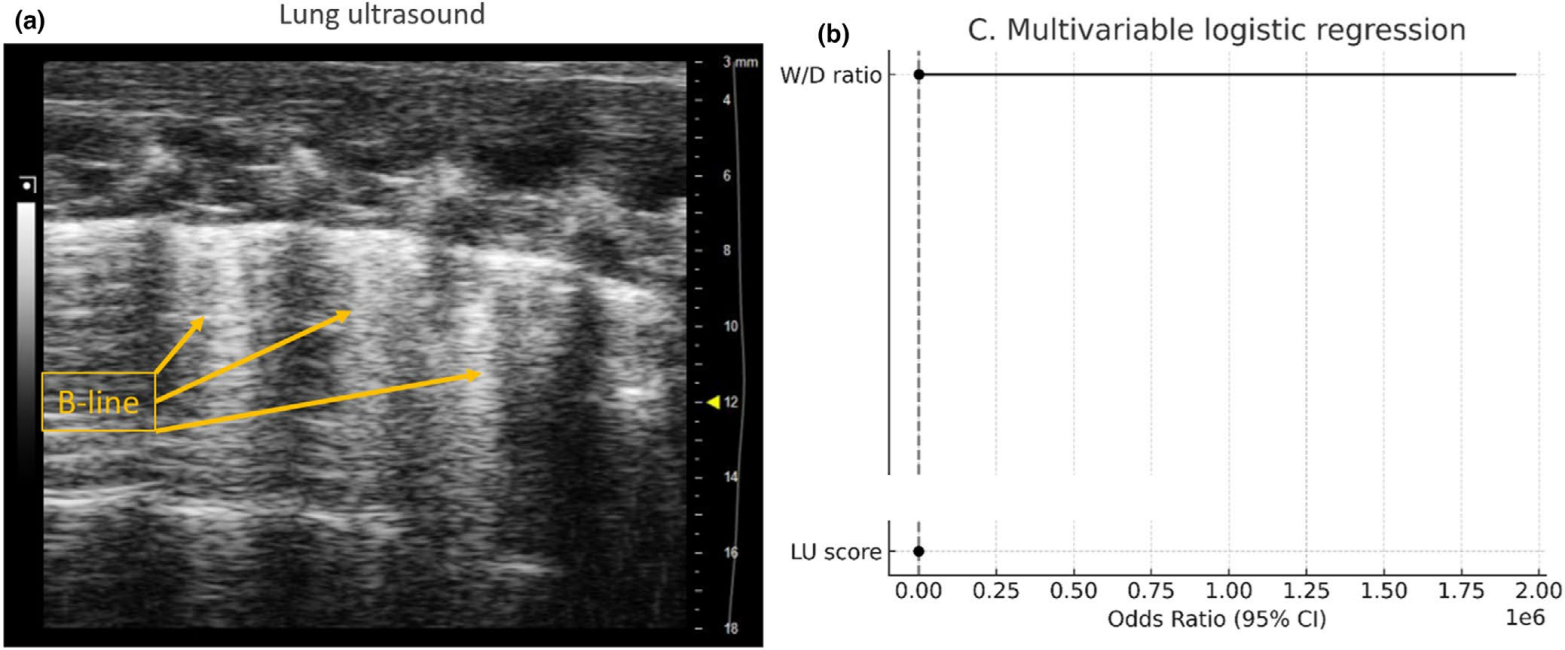
Diagnostic performance of the LU score and W/D ratio for detecting lung injury. (a) Representative lung ultrasound image showing B‐lines indicative of pulmonary congestion. (b) Forest plot of the multivariable logistic regression analysis including the LU score and W/D ratio, demonstrating that the LU score was independently associated with lung injury, whereas the W/D ratio showed a weaker association.

The results demonstrate that both LU scores and W/D ratios exhibited significant discriminatory ability, with ROC curves positioned well above the diagonal reference line. The LU score achieved a high AUC and detected lung edema effectively in this model. Although its AUC was higher than the W/D ratio, this should be interpreted cautiously, as the wet‐to‐dry ratio is the benchmark method in this experimental model. Consistently, the multivariable logistic regression analysis revealed that the LU score was independently associated with lung injury, whereas the W/D ratio showed a weaker and less precise effect. Therefore, the main conclusion is that LUS detects lung edema well and can be considered a useful non‐terminal tool for monitoring pulmonary congestion in rats (Figure 2).

**FIGURE 3:**
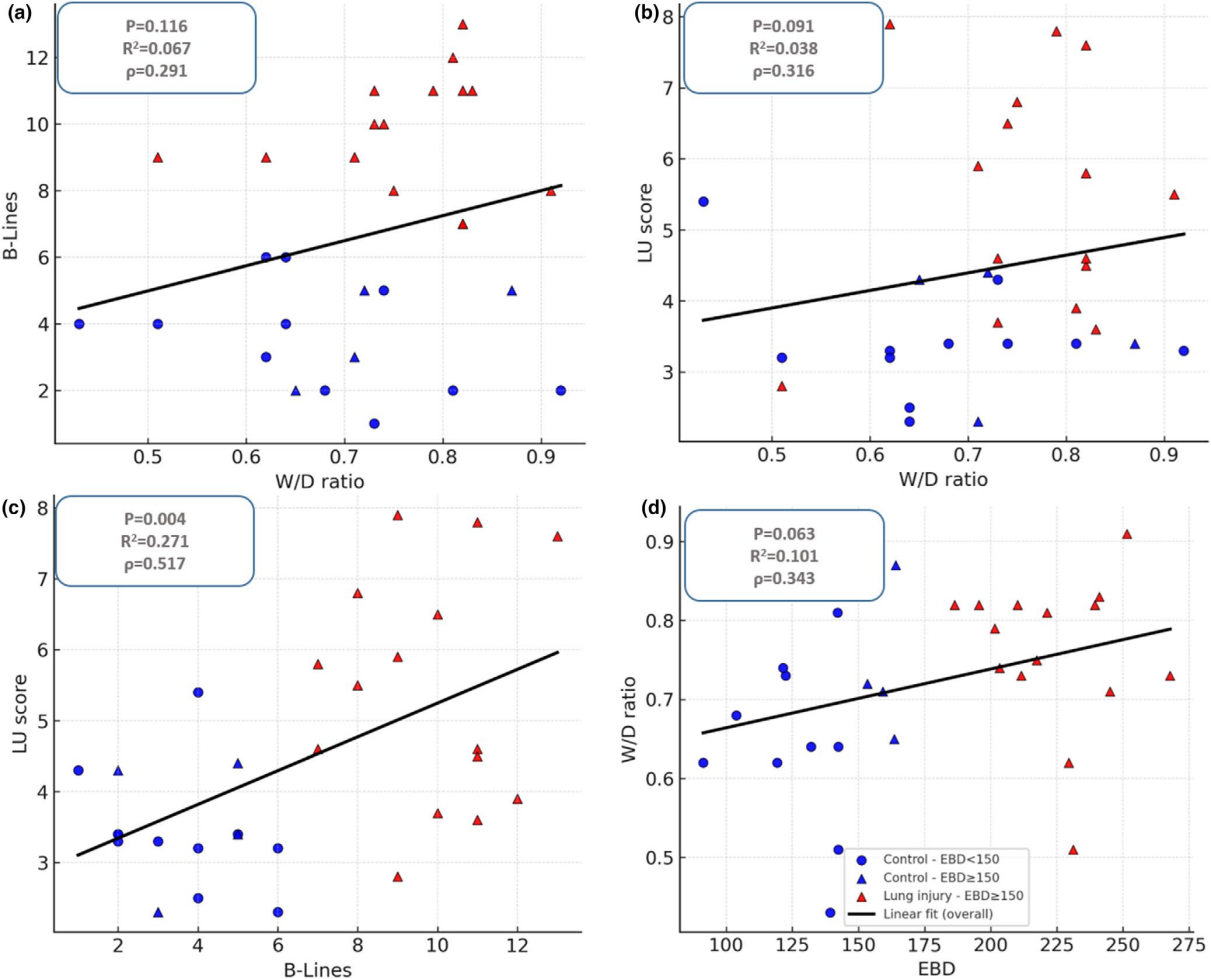
Correlations among pulmonary congestion markers. (a) Relationship between B‐Lines concentration and W/D ratio. (b) Relationship between LU score concentration and W/D ratio. (c) Relationship between LU score concentration and B‐Lines. (d) Relationship between W/D ratio and EBD. Control animals are represented by blue circles and lung‐injured animals by red triangles. Linear regression lines are shown for overall associations, with corresponding correlation coefficients (*R* ^2^) and *p*‐values indicated.

Analysis of correlations demonstrated that higher EBD concentrations were associated with an increased W/D ratio, a greater number of B‐lines, and higher LU scores, indicating consistent relationships between pulmonary extravasation and both gravimetric and ultrasound measures of congestion. In addition, the W/D ratio correlated positively with B‐lines and LU score, further supporting its role as a marker of pulmonary fluid accumulation. A strong association was also observed between B‐lines and LU score, reinforcing the validity of lung ultrasound as a reliable method for detecting pulmonary congestion. Although not all correlations reached statistical significance, the overall pattern consistently showed that worsening congestion was accompanied by concordant increases across all evaluated parameters (Figure 3).

**FIGURE 4:**
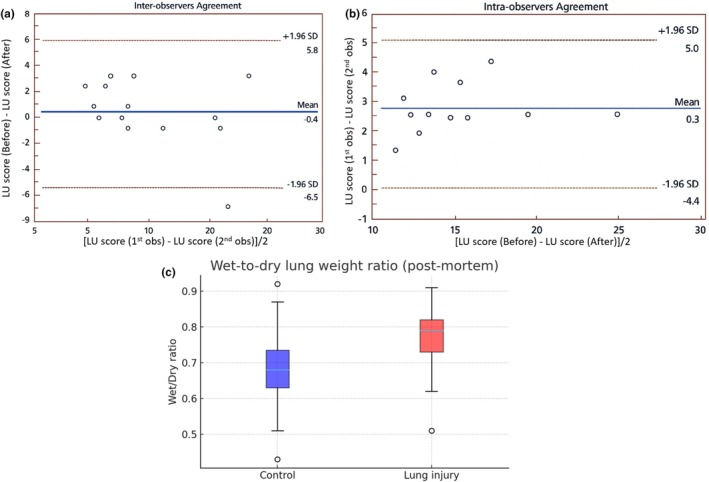
Bland–Altman plots evaluating the reproducibility of lung ultrasonography (LU) B‐line scores in rats. (a) Reproducibility of LU scores over time in 15 animals. (b) Interobserver agreement across 15 independent measurements. (c) Postmortem wet‐to‐dry lung weight ratio in control and lung injury groups.

Bland–Altman analysis showed good reproducibility of the LU score. Interobserver agreement presented a minimal mean bias (−0.4) with limits of agreement from −6.5 to 5.8, indicating no systematic differences between observers, while intraobserver agreement showed a mean bias close to zero (0.3) with limits from −4.4 to 5.0, confirming consistent reproducibility within the same observer. Together, these results support the reliability of B‐line quantification in this experimental model. In contrast, postmortem analysis of the wet‐to‐dry (W/D) lung weight ratio revealed higher values in the injury group compared with controls (mean 0.76 ± 0.10 vs. 0.69 ± 0.13; median 0.79 [0.73–0.82] vs. 0.68 [0.63–0.74]), reflecting increased lung fluid accumulation. Although the difference did not reach statistical significance (*t* = −1.82, *p* = 0.080), the moderate effect size (Cohen's *d* = 0.66) suggests a biologically relevant rise in lung water content. These findings indicate that while the W/D method detects a trend toward greater lung congestion, its terminal and less sensitive nature limits its utility compared with longitudinal monitoring by lung ultrasonography (Figure 4).

## 4 DISCUSSION

The present study highlights lung ultrasound (LUS) as a reliable and reproducible tool for quantifying pulmonary congestion, showing strong agreement with Evans Blue dye (EBD) extravasation and good diagnostic accuracy for detecting lung edema.

While pulmonary congestion in clinical settings typically arises from conditions such as renal failure or heart failure, in this preclinical rat model, oleic acid was used to induce acute pulmonary congestion. This resulted in characteristic changes of pulmonary edema, including increased relative lung weight (W/D ratio), greater EBD extravasation, and elevated pulmonary arterial pressure and pulmonary vascular resistance. These findings are consistent with previous studies employing the oleic acid model, in which the agent induces direct endothelial injury, increased capillary permeability, and extravascular fluid accumulation in the lung parenchyma (Matute‐Bello et al., 2008).

Concomitant with lung injury, this study also demonstrated a reduction in left ventricular diastolic diameter, accompanied by thickening of the interventricular septum and free wall, suggesting acute remodeling secondary to pressure overload. Similar alterations have been reported in experimental models of acute respiratory distress syndrome, where pulmonary congestion contributes to secondary cardiac dysfunction (Mekontso Dessap et al., 2016). In addition, oleic acid–induced injury was not limited to structural remodeling but also extended to functional changes, reflected in diastolic dysfunction parameters such as reduced E wave and e', confirming impaired ventricular filling. These findings align with reports indicating that pulmonary congestion extends beyond the alveolar compartment and significantly impacts cardiopulmonary coupling (Dessap et al., 2012).

The higher number of B lines and elevated LUS scores observed here corroborate both clinical and experimental studies that validate LUS as an early and sensitive marker of extravascular lung water (Volpicelli et al., 2012). Furthermore, the observed decrease in cardiac index and reduced TAPSE, along with an increased tricuspid E/e' gradient, reinforce the presence of right ventricular overload in acute pulmonary hypertension, consistent with similar experimental findings (Repessé et al., 2015). Collectively, these results confirm that the oleic acid model integrates both pulmonary congestion and cardiovascular dysfunction, closely resembling clinical pathophysiology.

Assessment of pulmonary congestion in clinical practice remains challenging due to anatomical variability, comorbidities, and technical differences in image acquisition. In contrast, preclinical rat models provide controlled conditions, ensuring reproducibility of findings. Notably, the LUS score in this study demonstrated greater diagnostic accuracy than the W/D ratio, as shown by the AUC values (0.88 vs. 0.72). This reinforces the applicability of LUS as a sensitive, noninvasive tool for detecting lung edema in this model for detecting pulmonary congestion and edema, in line with prior work validating B‐line scoring as an early marker of fluid overload and increased permeability (Volpicelli & Bouhemad, 2023).

Multivariate logistic regression further indicated that the LUS score remained an independent predictor of lung injury, while complementing conventional techniques such as the W/D ratio by offering superior sensitivity and real‐time applicability. Unlike gravimetric (W/D) or EBD methods, which are terminal and postmortem, LUS allows dynamic monitoring of congestion both before and after sacrifice.

Correlation analysis confirmed that both the LUS score and the number of B‐lines were significantly associated with pulmonary edema, supporting the sensitivity of lung ultrasound for detecting extravascular lung water. Jambrik et al. (2010) reported a strong correlation between the number of B‐lines and the wet/dry ratio in pigs with oleic acid–induced lung injury (*r* = 0.91; *p* < 0.001), indicating that LUS directly reflects pulmonary fluid accumulation. Similarly, Enghard et al. (2015). found a high correlation between LUS score and extravascular lung water index in critically ill patients (*r* = 0.91; *p* < 0.0001). Zhao et al. (2015) demonstrated that LUS scores were strongly associated with EVLWI in ARDS patients (*r* ^2^ ≈ 0.91). Anile et al. (2017) confirmed that a simplified LUS approach significantly correlated with EVLW index measured by transpulmonary thermodilution. Collectively, these studies validate LUS as a noninvasive and reliable tool for assessing pulmonary edema, even at early stages when conventional methods may lack sensitivity.

Thus, the stronger correlations observed between LU score/B lines and EBD, compared with the W/D ratio, reinforce the superiority of ultrasound as a noninvasive predictor of pulmonary congestion. Bland–Altman analysis confirmed good interobserver and intraobserver reproducibility, with mean differences near zero and narrow limits of agreement, underscoring the robustness of LUS as an assessment tool. These findings are consistent with studies demonstrating high reproducibility of B‐line detection across different examiners (Agricola et al., 2005; Volpicelli et al., 2012). Finally, gravimetric analysis validated the injury model, confirming higher lung water content and establishing a direct link between histopathology and ultrasound measurements.

Taken together, the present findings demonstrate that LUS is a sensitive, reliable, and reproducible tool for detecting and semi‐quantifying pulmonary edema in rats. The LUS score showed a strong correlation with the wet‐to‐dry lung weight ratio, indicating that lung ultrasound detects pulmonary edema well in this experimental model. LUS provides a practical non‐terminal method for monitoring pulmonary congestion but does not replace gravimetric measures. Importantly, while EBD extravasation reflects vascular permeability, it does not directly quantify total lung water; therefore, our analysis and conclusions were revised to emphasize the relationship between LUS and gravimetric measures of edema. This refinement better aligns with the physiological basis of pulmonary congestion assessment in experimental models.

Although EBD extravasation is widely used as a reference for pulmonary vascular leak, it reflects increased endothelial permeability rather than the total volume of lung water. Therefore, while the correlation between LUS findings and EBD supports the sensitivity of ultrasound to detect permeability‐related congestion, EBD cannot be considered a direct surrogate for the extent of pulmonary edema. This distinction is particularly relevant in hydrostatic forms of edema, where lung water content may rise without concomitant increases in EBD.

## 5 LIMITATIONS

Despite the findings, this study is limited by the relatively small sample size, the potential effects of anesthesia on cardiopulmonary physiology, the reliance on a terminal method (wet‐to‐dry ratio) for comparison, and variability associated with differences in operator training and ultrasound expertise.

## 6 CONCLUSION

In this experimental rat model of oleic acid‐induced lung injury, lung ultrasonography proved to be a reliable and reproducible tool for quantifying pulmonary congestion, showing strong agreement with the wet‐to‐dry lung weight ratio and demonstrating an excellent ability to detect and semi‐quantify pulmonary edema noninvasively. The LU score demonstrated minimal inter‐ and intraobserver bias, supporting its robustness for longitudinal assessment, while the W/D method was limited by its terminal nature and lower sensitivity. These findings highlight LU as a practical, non‐invasive alternative for evaluating extravascular lung water in small‐animal research, with potential translational relevance for monitoring pulmonary congestion in preclinical and settings.

## FUNDING INFORMATION

This research received no specific funding from public, commercial, or non‐profit organizations.

## CONFLICT OF INTEREST STATEMENT

The author declare no conflicts of interest.

## Data Availability

All data and materials related to this manuscript are available through the medical school database at: https://www.gedweb.com.br/usp.
